# Influence of Muscle Mass and Outdoor Environmental Factors on Appetite and Satiety Feeling in Young Japanese Women

**DOI:** 10.3390/ijerph15010167

**Published:** 2018-01-21

**Authors:** Masahiro Okada

**Affiliations:** Department of Food and Dietetics, Hiroshima Bunka Gakuen Two-Year College, 3-5-1 Nagatsukanishi, Asaminami-ku, Hiroshima 731-0136, Japan; okada@hbg.ac.jp

**Keywords:** satiety, food intake, muscle mass, outdoor environmental factor, outdoor temperature

## Abstract

Research on the influence of relationships among satiety, muscle mass, and outdoor environmental factors is sparse. In this work the relationships among satiety feeling, body composition, and outdoor environmental factors on eating in healthy young Japanese women are investigated. Fifty three (53) women were examined over an approximately 2-year period. All participants ate the same lunch; feelings of satiety and body composition were measured before and immediately after lunch. Satiety was assessed using a visual analog scale. Outdoor environmental factors were recorded at the time of measurement. Results showed that satiety before lunch decreased with increased muscle mass and decreased humidity (*p* < 0.05). The Δ satiety increased on eating with increased outdoor temperature (*p* < 0.05). The Δ satiety with high outdoor temperature was significantly greater than with low outdoor temperature (*p* = 0.005). Decreased muscle mass more influenced Δ satiety with respect to outdoor temperature than increased muscle mass (*p* = 0.007). The results suggest that increased muscle mass and decreased humidity increase hunger (unlike satiety) before eating. The findings also show that outdoor temperature clearly influences the magnitude of satiety on eating. Increasing muscle mass may be useful for satiety control at various outdoor temperatures in young women.

## 1. Introduction

Satiety may be characterized as sensitivity fullness and by how quickly an individual feels full after eating; or it could be characterized as to how long a person feels full before they want to start eating again [1,2]. Satiety responsiveness is a reflection of human appetite. Studies of appetite have investigated the relationship between satiety and body composition [3,4]. Fat mass and skeletal muscle mass are important factors in appetite regulation [5,6,7]. Reduction of fat mass in overweight and obese women results in increased appetite and decreased fullness [8]. Interestingly, studies have implicated fat-free mass and skeletal muscle mass in appetite regulation [7,8]. A recent study of children showed that muscle mass is predictive of decreases in satiety responsiveness; fat mass is predictive of increases in food responsiveness and desire to eat [2]. Studies have also suggested that muscle mass increases energy needs, which results in upregulation of appetite [4,5,9]. However, further clarification is required about the relationship between muscle mass and fat mass and appetite regulation. 

Many ambient factors, such as color, temperature, and sound, influence the intake of food [10] and satiety responsiveness [11]. In particular, outdoor temperature is considered a strong influencing factor on satiety before and after food intake [12,13]; that is a matter the author addressed in a previous study [14]. In the present study, satiety before and after eating among healthy young women was measured; body composition and outdoor temperature, relative humidity, atmospheric pressure, and day length were determined at the same time between 2010 and 2012. However, it is necessary to understand the relationship between satiety and outdoor environmental factors—both before and after food intake.

It is evident that body composition and outdoor environmental factors are associated with appetite. However, to the best of the author’s knowledge, the relationship among body composition, outdoor environmental factors, and satiety on appetite have not been sufficiently examined. Few studies have investigated the relationships among body composition and outdoor environmental factors on satiety before and after eating. To clarify those relationships, previously unanalyzed raw data from studies from 2010 to 2012 was examined, original findings were developed and new suggestions made from the results.

## 2. Materials and Methods 

### 2.1. Study Population

In all, 53 healthy female Japanese university students volunteered to participate. The Human Studies Committees of Hiroshima Bunka Gakuen Two-Year College approved the study. Informed consent was obtained from all participants. None of the young women were smokers, menstruating, taking prescription medications, or had a history of serious diseases, such as cancer, cardiovascular disease, and diabetes. All the participants were interviewed and confirmed that they were in good health, without strong stress, without anxiety about food in pre-menstruation, were getting sufficient sleep, and had fasted from breakfast until lunch. What the participants had eaten for breakfast was also recorded and it was confirmed that no alcohol, caffeine, or capsaicin had been consumed. The participants were asked not to consume alcohol for a period of at least 24 h before the measurements were taken. Hydration intake was restricted from breakfast until the pre-measurement. All participants were asked not to undertake moderate or vigorous exercise over a period of at least 12 h until the measurements were taken. The data were collected over an approximately 2-year period (March 2010 to April 2012). 

### 2.2. Food Intake and Measurements

Each participant underwent measurements only on one day (holiday or non-working day). All the measurements were taken at least 3 h after breakfast, with the participants wearing light indoor clothing. The measurements were made individually with the participants alone in a quiet room with appropriate ventilation and lighting. The room temperature was maintained at 20.3 ± 0.9 °C, and all the measurements were taken after the participants had adapted to the room temperature for 1 h. All the participants went to the toilet before the measurements.

Before measuring the satiety data, weight and height were measured with the participants wearing light indoor clothing with emptied pockets and without shoes and socks. Body composition measurement was conducted using foot-to-foot bioelectrical impedance analysis in a standing position. Body fat percentage and muscle mass were determined using a BC-520 body composition meter (Tanita Corporation, Tokyo, Japan). The body composition was measured after the participants confirmed the restrictions and conditions detailed in Section 2.1.

I obtained the satiety data between the hours of 11:30 and 12:00 (before lunch and immediately after lunch). A lunch of gyudon (beef bowl), consisting of rice and beef (total energy, 3372 kJ; 66.4% carbohydrate, 12.8% protein, 20.8% fat, and 790 mg sodium), was provided to all participants. The meal contained no caffeine or capsaicin. Satiety was assessed using a visual analog scale (100 mm) before and immediately after lunch [14,15,16]. The numerical value of 0–100 mm was described as ranging from “I feel no satiety at all” (hunger) to “I have a strong feeling of satiety” (full).

### 2.3. Outdoor Environmental Factors 

Details of outdoor temperature, atmospheric pressure, relative humidity, and day length were obtained from the Hiroshima Local Meteorological Observatory, located approximately 3 km from the study location. The mean values of the environmental data were obtained between 11:30 and 12:00 on the day of the measurements.

### 2.4. Data Analysis

All data were analyzed using SPSS for Windows, version 17.0 (IBM SPSS, Tokyo, Japan) and Interaction! software (http://www.danielspper.com/Interaction/). Descriptive statistics for all participants and outdoor environmental factors were expressed as means ± standard deviation (SD) or standard error (SE). Paired *t* tests were used to compare satiety before and after lunch in all participants. Multiple regression analysis was undertaken to characterize the relationships among body composition, outdoor temperature, and satiety before and after lunch. One-way analysis of variance (ANOVA) was used to compare Δ satiety of participants stratified by outdoor temperature (cold, <15.0 °C; mild, 15.0°–25.0°; hot, >25.0°). A simple slopes test was used to show the interaction among participant muscle mass (mean, +1 SD, and −1 SD), Δ satiety, and outdoor temperature [17].

## 3. Results

Table 1 shows the participants’ characteristics (*n* = 53) and outdoor environmental factors. The age (mean ± SD) of participants was 20.4 ± 2.6 years (range, 18–29 years), height 1.6 ± 0.1 m (range, 1.5–1.7 m), and weight 52.0 ± 7.4 kg (range, 41.7–72.6 kg). Mean body fat percentage was 28.6 ± 5.2% (range, 17.4–40.6%), and muscle mass was 34.5 ± 3.0 kg (range, 28.8–42.2 kg). The mean outdoor temperature was 18.1 ± 9.0°C (range, 1.3–31.6°), atmospheric pressure 1007.1 ± 6.8 hPa (range, 994.0–1022.4 hPa), relative humidity 67.0 ± 10.9% (range, 41.0–89.0%), and day length 5.4 ± 4.2 h (range, 0–13 h).

Table 2 shows the mean satiety values before and immediately after lunch and Δ satiety. Mean values of satiety before lunch were 45.0 ± 17.3 mm (range, 0.0–84.0 mm) and satiety immediately after lunch 89.8 ± 7.6 mm (range, 65.0–100.0 mm). There was a significant difference between satiety before lunch and immediately after lunch (*p* < 0.001). The mean Δ satiety was 44.7 ± 16.7 mm (range, 11.0–100.0 mm).

Table 3 shows the relationships among satiety and factors of body composition and outdoor environment. Decreased satiety before lunch was associated with increased muscle mass (standard regression coefficient [β] = −0.784, *p* = 0.030). Increased satiety before lunch was associated with increased relative humidity (β = 0.299, *p* = 0.033). No relationship between satiety immediately after lunch and those factors was observed. However, increased Δ satiety was associated with increased outdoor temperature (β = 0.145, *p* = 0.025).

**Figure 1:**
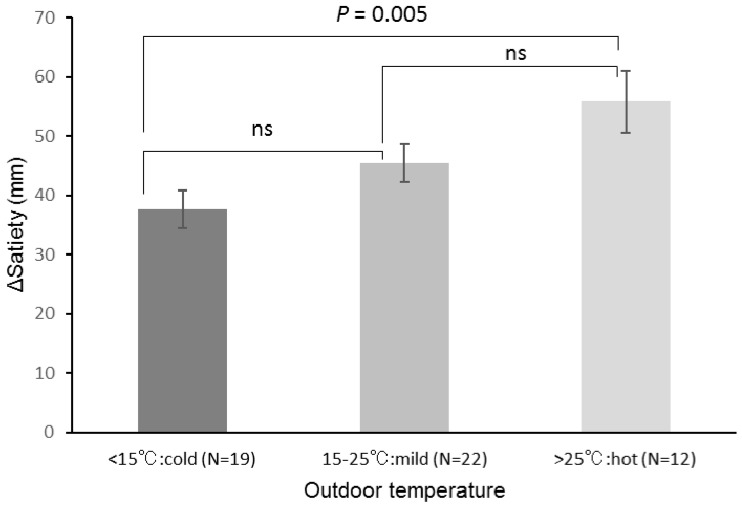
Comparison of Δ satiety at stratified outdoor temperatures. Δ satiety of participants was stratified into three groups by outdoor temperature (<15 °C, cold; 15 °C–25 °C, mild; >25 °C, hot). Values are means ± standard errors. *p* values based on one-way ANOVA in stratified outdoor temperature groups.

Figure 1 shows comparisons of Δ satiety stratified by outdoor temperature (cold, <15.0 °C; mild, 15.0 °C–25.0 °C; hot, >25.0 °C). There were significant differences in Δ satiety between hot and cold (*p* = 0.005), but not between hot and mild and mild and cold. 

**Figure 2:**
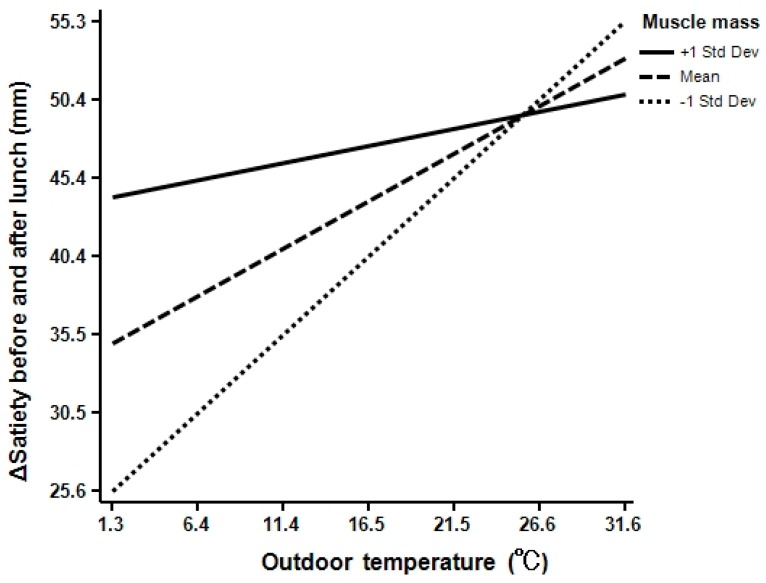
Interaction among Δ satiety, outdoor temperature, and muscle mass. +1 SD muscle mass (simple slope, 0.214; *p* = 0.576), mean muscle mass (simple slope, 0.599; *p* = 0.017), −1 SD muscle mass (simple slope, 0.983; *p* = 0.007).

The simple slopes test revealed interactions among Δ satiety, outdoor temperature, and muscle mass. The Δ satiety interacted significantly with outdoor temperature and −1 SD muscle mass (simple slope, 0.983; 95% confidence interval [Cl], 0.278–1.688; *p* = 0.007), mean muscle mass (simple slope, 0.599; 95% Cl, 0.110–1.088; *p* = 0.017), and +1 SD muscle mass (simple slope, 0.214; 95% Cl, −0.551–0.980; *p* = 0.576) (Figure 2). 

## 4. Discussion

Many observational studies of appetite, including hunger and satiety as related to obesity and the endocrine system, have been undertaken [3,4,8,18,19,20]. In investigations of obesity, weight loss has been reported to induce an increase in hunger and a desire to eat [8]. With body composition, it has been found that the daily hunger sensation level is positively correlated with free fat mass and negatively correlated with fat body mass [4,9]. Furthermore, it has been suggested that appetite sensation is associated with weight loss, including body fat loss [20]. When appetite was controlled separately from fat mass, it was also reported that fat free mass is positively associated with self-determined meal size and daily energy intake [4]. Recently, Steinsbekk et al. showed that muscle mass predicts a decrease in satiety responsiveness and fat mass predicts an increase in food responsiveness in childhood. From these results, the relationship between appetite and body composition is evidently important [2]. 

In addition, satiety should be significantly related to body composition. In the present study, participants were questioned about satiety feelings before and after lunch. By questioning about satiety rather than hunger before lunch, a more accurate analysis of the relationships of postprandial sensation than in a previous study [14] was performed. In particular, satiety after eating tends to be less accurate because the baseline of each participant is different [16]. In this study, Δ satiety was adopted as the magnitude of satiety: the indicator of strong or weak satiety.

Increased muscle mass significantly decreased satiety before lunch. In addition, increased body fat percentage increased satiety before lunch, but not significantly. This result seems to explain that hunger is increased by increased free fat mass and muscle mass, as previous studies have also shown [2,21,22]. Increasing muscle mass increases energy intake, suggesting that it is strongly related to satiety—especially before eating (hunger sensation). The relationship between body composition and satiety after eating or Δ satiety was expected; however, the relationship was not strong. If satiety after eating and Δ satiety decrease, it is believed that appetite may continue during eating. This persistence of appetite is very important in appetite and obesity research. Further research is necessary to determine the relationship between satiety after eating and valance of body composition.

Although appetite control is thought to be related to environmental factors, the details of the relationship are unknown. In particular, ambient temperature affects basal metabolic rate, caloric consumption, and energy expenditure. In general, hunger, satiety, and appetite should be influenced by changes in ambient temperature: cold temperatures induce increased basal metabolic rate and high-energy consumption [11,12,13]. However, previous studies have not found a difference in hunger and satiety with short-term temperature changes. 

In the present study, the relationship between participants’ feelings of satiety before and after eating using outdoor environmental data obtained from a meteorological observatory was analyzed. It was found that increased satiety before eating was affected by increased relative humidity rather than outdoor temperature. This result indicates that hunger before eating increases with decreased relative humidity. Ambient temperature and energy expenditure have a negative correlation; however the relationship with hunger is not clear, and there is insufficient information on the relationship between humidity and hunger [23]. In the present research, it was found that the reason for decreasing hunger could be discomfort caused by high humidity. In particular, the outdoor temperature in Hiroshima is very high in summer. If the humidity rises, people feel discomfort and lose hunger [24]. In autumn and winter, the outdoor temperature is lower and it is less humid; energy expenditure and cold stress rather than discomfort may influence hunger [11]. In studies of hunger before eating, consideration should be made of the influence of outdoor temperature and humidity as well as of seasonal environmental conditions.

With regard to satiety, it was found that Δ satiety before and after eating is influenced by outdoor temperature. Decreased outdoor temperature reduces Δ satiety. It is considered that decreased Δ satiety leads to a weak satiety sensation and strong continuous appetite. As a result, a person may increase food intake with cold outdoor temperatures. Indeed, this study identified a clear difference between cold and hot conditions on Δ satiety. In a previous study, the author found that outdoor temperature influenced Δ α-amylase activity and Δ low-to-high frequency ratio of heart rate variability after eating. In addition, the autonomic nervous system or stress of outdoor temperatures may influence the feeling of satiety [14]; that may be related to rhythmicity of food intake with respect to seasonality [25]. Even though indoor conditions may be constant, outdoor environmental conditions and the season may influence the rhythmicity of a person’s appetite. The mechanism of ∆ satiety feeling due to outdoor temperature may not be explained by short-term energy expenditure and appetite-related hormone response [11,26]. To explain this result, it may be necessary to investigate the relationship between human rhythms and the accumulation of outdoor temperatures over a long period. 

In this study, interesting results concerning the interaction among satiety feeling, outdoor temperature, and muscle mass were obtained. As the muscle mass increased, there was almost no influence on the satiety feeling due to outdoor temperature. However, when the muscle mass decreased, the satiety feeling was strongly influenced by the outdoor temperature. The results are not shown, but no interaction between fat percentage and outdoor temperature regarding satiety feeling was found. This result suggests that the muscles help to regulate satiety feeling arising from various outdoor temperatures. Conversely, if there is a decrease in muscle mass, it may not be possible to control appetite under various outdoor temperatures. It is suggested that when people adapt to various environments, muscles are one of the most important organs in appetite control. 

This study has several limitations. First, the participants were healthy young women living in Hiroshima. Body composition is generally different between women and men. Women may also have a different physiological and psychological response to climate from men. Therefore, these results may not be generalizable to all populations [27]. Second, the participants ate the same food. If participants overeat ad libitum, energy balance and physiology variables may strongly influence satiety [28]. In addition, the amount of fat and carbohydrate contained in the food may influence satiety [29]. Third, seasonality could not be fully taken into account. In Hiroshima, there are four seasons, but seasonal factors were not accommodated. Of the recordings, 19 were made in autumn and winter (36%) and 34 in spring and summer (64%). The diurnal rhythm associated with seasonal change is considered a strong influence on the feeling of satiety [25,30]. Fourth, this study was observational, and it could not examine hormonal mechanisms, such as leptin, ghrelin, and peptide YY, with respect to satiety [30,31]. In addition, food intake and appetite may relate to several myokines secreted from muscle [32]. Fifth, the participants had adequate sleep prior to measurements, but lifestyle rhythms, including participants’ sleep habits, could not be considered. In particular, circadian rhythms related to sleeping habits are believed to strongly influence satiety [33,34]. Sixth, although air-displacement plethysmography or dual-energy X-ray absorptiometry may be recommended for measuring body composition, the type of bioelectrical impedance analysis machine used in the present study has been used in over 200 studies [35]. Furthermore, the author could not discuss the correlation between the feeling of Δ satiety and brain activity. There is a limit with using only the visual analog scale method. In future, it will be necessary to consider brain research [16,22]. Despite these limitations, new relationships in satiety responsive to changes in muscle mass and outdoor environmental factors were found. 

## 5. Conclusions

Increased muscle mass and decreased relative humidity reduce satiety before eating. Therefore, increased muscle mass and decreased relative humidity may increase hunger before eating. The magnitude of satiety after eating is reduced by decreased outdoor temperature. It is suggested that decreased magnitude of satiety may lead to continuous appetite and a desire to eat. Furthermore, women with high muscle mass are less susceptible to changes in satiety from outdoor temperatures. It is considered that increasing muscle mass may be useful for satiety control related to various outdoor temperatures in young women. Satiety and food intake are important with respect to appetite, and their regulation is thought to be important for human health. Hitherto, however, there has been little information about the relationship between satiety and environmental factors. The present findings may offer a better understanding of the regulation of human appetites in various environments.

## Figures and Tables

**Table 1:** Characteristics of the study population and outdoor environmental factors (*n* = 53).

Characteristic	Mean ± SD	Range
Age (years)	20.4 ± 2.6	18–29
Height (m)	1.6 ± 0.1	1.5–1.7
Weight (kg)	52.0 ± 7.4	41.7–72.6
Body fat percentage (%)	28.6 ± 5.2	17.4–40.9
Muscle mass (kg)	34.5 ± 3.0	28.8–42.2
Outdoor environmental factors		
Outdoor temperature (°C)	18.1 ± 9.0	1.3–31.6
Atmospheric pressure (hPa)	1007.1 ± 6.8	994.0–1022.4
Relative humidity (%)	67.0 ± 10.9	41.0–89.0
Day length (h)	5.4 ± 4.2	0–13

SD = standard deviation.

**Table 2:** Changes in satiety before and after lunch.

Variable (mm)	Mean ± SD	Range
Satiety before lunch	45.0 ± 17.3	0.0–84.0
Satiety immediately after lunch	89.8 ± 7.6 ***	65.0–100.0
Δ Satiety before and after lunch	44.7 ± 16.7	11.0–100.0

SD = standard deviation; *p* values were determined using the paired *t* test. *** p < 0.001 vs. before lunch.

**Table 3:** Relationships among satiety before and after lunch and various factors.

Variable Factors	Satiety before Lunch	Satiety Immediately after Lunch	Δ Satiety Before and after Lunch
Body fat percentage (%)	0.393 (0.120)	−0.275 (0.290)	−0.183 (0.117)
Muscle mass (kg)	−0.784 (0.030)	0.227 (0.546)	0.223 (0.201)
Environmental factors			
Outdoor temperature (°C)	−0.236 (0.095)	0.235 (0.102)	0.145 (0.025)
Atmospheric pressure (hPa)	−0.003 (0.984)	−0.196 (0.177)	−0.089 (0.167)
Relative humidity (%)	0.299 (0.033)	0.041 (0.778)	−0.020 (0.764)
Day length (h)	−0.237 (0.104)	0.002 (0.987)	0.033 (0.635)

Analysis was adjusted for room temperature, age, height, and weight. β = standardized coefficient. *p* values appear in parentheses. Δ satiety before and after lunch was adjusted by adding the satiety before lunch.
